# Associations of non-HPV genital pathogen detection with HPV positivity and cervical findings in women undergoing screening in Xinjiang, China: a retrospective cross-sectional study

**DOI:** 10.3389/fmed.2026.1919155

**Published:** 2026-09-09

**Authors:** Xiaolu Yu, Weilan He, Xiumin Ma, Yiliminuer Keremu, Xiaoqian Shang, Weina Kong, Chen Lin, Fan Guo

**Affiliations:** 1 Department of Medical Laboratory Center, Xinjiang Key Laboratory of Molecular Biology for Endemic Diseases, Tumor Hospital Affiliated to Xinjiang Medical University, Urumqi, Xinjiang, China; 2 Department of Clinical Laboratory, The Second Hospital of Shanxi Medical University, Taiyuan, China; 3 College of Basic Medical Sciences, Xinjiang Key Laboratory of Molecular Biology for Endemic Diseases, Xinjiang Medical University, Urumqi, Xinjiang, China

**Keywords:** chlamydia trachomatis, genital diseases, papillomavirus infections, sexually transmitted diseases, uterine cervical dysplasia

## Abstract

This study examines the associations between various non-HPV genital tract pathogens—including Chlamydia trachomatis (CT), Neisseria gonorrhoeae (NG), Ureaplasma urealyticum (Uuu), Ureaplasma parvum (Uup1, Uup3, Uup6, Uup14), Mycoplasma hominis (MH), Mycoplasma genitalium (MG), and Herpes simplex virus type II (HSV-2)—and HPV positivity and cervical abnormalities. Furthermore, it examines the co-detection patterns of these pathogens and human papillomavirus (HPV) in relation to cervical cytological and histopathological findings. A retrospective cross-sectional study was conducted involving 154 HPV-positive women and 193 HPV-negative women who underwent cervical cancer screening at our gynecology outpatient clinic during the study period. All participants underwent multiplex genetic testing for cervical pathogens. The six most prevalent HPV subtypes identified were HPV16, 53, 52, 58, 51, and 33. Detection of Uuu, Uup1, Uup6, MH, and CT was significantly associated with HPV positivity. In conclusion, selected non-HPV genital pathogens were associated with HPV positivity and concurrent cervical cytological abnormalities, whereas no significant association was identified with histopathologically confirmed cervical SILs among HPV-positive women.

## 1 Introduction

Human papillomavirus (HPV) infection is a significant factor contributing to cervical lesions in women. While HPV infection is a necessary cause of cervical cancer, it is not sufficient on its own (1). Genetic factors may influence both susceptibility to and the persistence of HPV infection (2). During physical examinations, it is crucial to assess microbial indicators in vaginal secretions, as well as the presence of HPV and sexually transmitted disease pathogens in cervical secretions, to ensure women's cervical health (3). The levels of hormones, inadequate personal hygiene practices, and sexually transmitted infections may disrupt the microbial homeostasis of the female reproductive tract, potentially altering the cervical microenvironment (4). The composition and stability of the vaginal microbiota are closely related to local mucosal health (5). Several biological mechanisms have been proposed to explain the observed associations between non-HPV genital pathogens and HPV-related cervical abnormalities. Alterations in the vaginal and cervical microbial environment may promote local inflammatory responses, impair epithelial barrier integrity, and modify mucosal immune function (5, 6). Persistent local inflammation and dysregulated antiviral immune responses may, in turn, interfere with effective HPV clearance and favor viral persistence, while prolonged epithelial inflammation may be associated with the development of cervical cytological abnormalities (7, 8). Conversely, HPV-related epithelial and immune changes may also modify the cervical microenvironment and facilitate the persistence or detection of other genital microorganisms. Therefore, the relationship between HPV and non-HPV genital pathogens may be bidirectional, and the underlying mechanisms remain incompletely understood.

Although associations between HPV and other genital pathogens have been reported in different populations, regional evidence from women undergoing cervical cancer screening in Xinjiang remains limited. Whether the patterns of non-HPV genital pathogen detection and their associations with HPV positivity and cervical abnormalities observed in other populations are also present in women screened in Xinjiang has not been sufficiently characterized. Moreover, few regional clinical data have simultaneously evaluated non-HPV genital pathogen detection in relation to HPV positivity, cervical cytological categories, and histopathological findings within the same screening population. Therefore, the present study aimed to characterize the distribution of selected non-HPV genital pathogens and HPV genotypes in women undergoing cervical cancer screening in Xinjiang and to examine their associations with HPV positivity, cervical cytological abnormalities, and available histopathological findings.

## 2 Materials and methods

### 2.1 Study subject

A total of 154 HPV-infected women who underwent cervical cancer screening at the Gynecological Oncology Hospital in Urumqi, Xinjiang, from January to December 2025 were included in the observation group, with an average age of 48.1 years. A total of 193 HPV-negative women were included in the HPV-negative group, with a mean age of 48.3 years. Eligibility criteria included a history of sexual intercourse, reproductive age, no systemic antibiotic treatment within the previous 2 weeks, no recent use of sex hormone medications, no recent vaginal douching, and no history of chemotherapy before sampling. Pregnant or lactating women and women with autoimmune diseases, malignancies, or mental disorders were excluded.

Detailed individual-level information on sexual behavior beyond a history of sexual intercourse, parity, contraceptive use, smoking status, and other immunocompromising conditions was not available in the study dataset.

### 2.2 Research methodology

#### 2.2.1 Cervical cytology examination

Patients were positioned in the lithotomy position, and a vaginal speculum was employed to expose the cervix. First, a cotton swab was utilized to clean the secretions from the cervical os. Next, a cervical brush was rotated clockwise five times to a depth of 2 cm within the cervical os. Finally, the cervical brush was slowly withdrawn to ensure an adequate collection of exfoliated epithelial cells from both the cervical os and canal. The head of the sampling brush was placed into a vial containing Cytorich preservation fluid, which was labeled with the subject's identification number. After specimen preparation, cervical cytological findings were interpreted with reference to The Bethesda System for Reporting Cervical Cytology (TBS) (9). For statistical analysis, participants were classified according to the cytological results recorded in the study database into three groups: no epithelial cell abnormality, lower-grade cervical cytological abnormalities, and higher-grade cervical cytological abnormalities.

#### 2.2.2 Cervical pathology examination

Women requiring further diagnostic evaluation underwent colposcopic examination with cervical tissue sampling and/or endocervical curettage for histopathological examination. According to the histopathological results recorded in the study database, the findings were categorized into three groups: normal histopathology, inflammatory changes, and cervical squamous intraepithelial lesions (SILs). These histopathological categories were analyzed separately from the cervical cytological categories described in Sec. 2.2.1.

#### 2.2.3 HPV genotyping test

The HPV genotyping kit was obtained from Guangdong Hybribio Biotechnology Co., Ltd. The assay used PCR amplification combined with flow-through hybridization and detected 21 HPV genotypes, including HPV16, 18, 31, 33, 35, 39, 45, 51, 52, 53, 56, 58, 59, 66, 68, 6, 11, 42, 43, 44, and 81. Fourteen genotypes were classified as high-risk HPV types: HPV16, 18, 31, 33, 35, 39, 45, 51, 52, 56, 58, 59, 66, and 68. DNA extraction was performed using the Roche MagNA Pure 96 system, amplification was performed using the Hybribio TC-96 system, and flow-through hybridization was performed using the HBHM-9000A system. All procedures were performed according to the manufacturer's instructions, and the required quality-control criteria were satisfied before test results were accepted. Assay-specific sensitivity and specificity were not independently evaluated in the present study, and verified numerical performance characteristics could not be retrospectively retrieved from the available study records.

#### 2.2.4 Pathogen ten-plex assay for reproductive tract infections

The multiplex genital pathogen detection kit was obtained from Guangdong Hybribio Biotechnology Co., Ltd. The assay detected MH, NG, Uup6, Uuu, CT, Uup3, Uup1, Uup14, MG, and HSV-2 using PCR amplification combined with flow-through hybridization. DNA extraction was performed using the Roche MagNA Pure 96 system, amplification was performed using the Hybribio TC-96 system, and flow-through hybridization was performed using the HBHM-9000A system. All procedures were performed according to the manufacturer's instructions, and the required quality-control criteria were satisfied before test results were accepted. The assay provided qualitative positive/negative results; quantitative PCR was not performed, and pathogen load was not measured. A positive result was defined as molecular detection of the corresponding pathogen nucleic acid in the cervical specimen. Because pathogen load, culture results, clinical symptoms, and longitudinal persistence were not assessed, pathogen positivity was interpreted as molecular detection rather than as confirmation of clinically active infection. Assay-specific sensitivity and specificity were not independently evaluated in the present study, and verified numerical performance characteristics could not be retrospectively retrieved from the available study records.

### 2.3 Statistical analysis

All statistical analyses were performed using SPSS version 25.0 and GraphPad Prism version 8.0. Categorical data were presented as numbers and percentages [n (%)], and between-group comparisons were performed using the chi-square test, with *P* < 0.05 considered statistically significant. Odds ratios (ORs) with 95% confidence intervals (CIs) were calculated to quantify the unadjusted associations between genital pathogen detection and HPV positivity. ORs were interpreted as measures of association rather than causation. Pathogens with no positive cases were excluded from OR estimation. Estimates based on very small numbers of pathogen-positive participants were considered exploratory and were interpreted cautiously because of their limited precision and statistical power. Because individual-level information on several potential confounding factors, including detailed sexual behavior, parity, contraceptive use, smoking status, and HPV vaccination status, was unavailable, multivariable-adjusted analyses could not be performed.

### 2.4 Ethics approval and consent to participate

This study was conducted in accordance with the ethical principles of the Declaration of Helsinki and approved by the Ethics Committee of the Affiliated Cancer Hospital of Xinjiang Medical University (Approval No. K-2024021). Written informed consent was obtained from all participants for the collection and use of specimens for research purposes, as well as for the publication of the data.

## 3 Results

### 3.1 Detection status of reproductive tract infection pathogens

**Figure 1:**
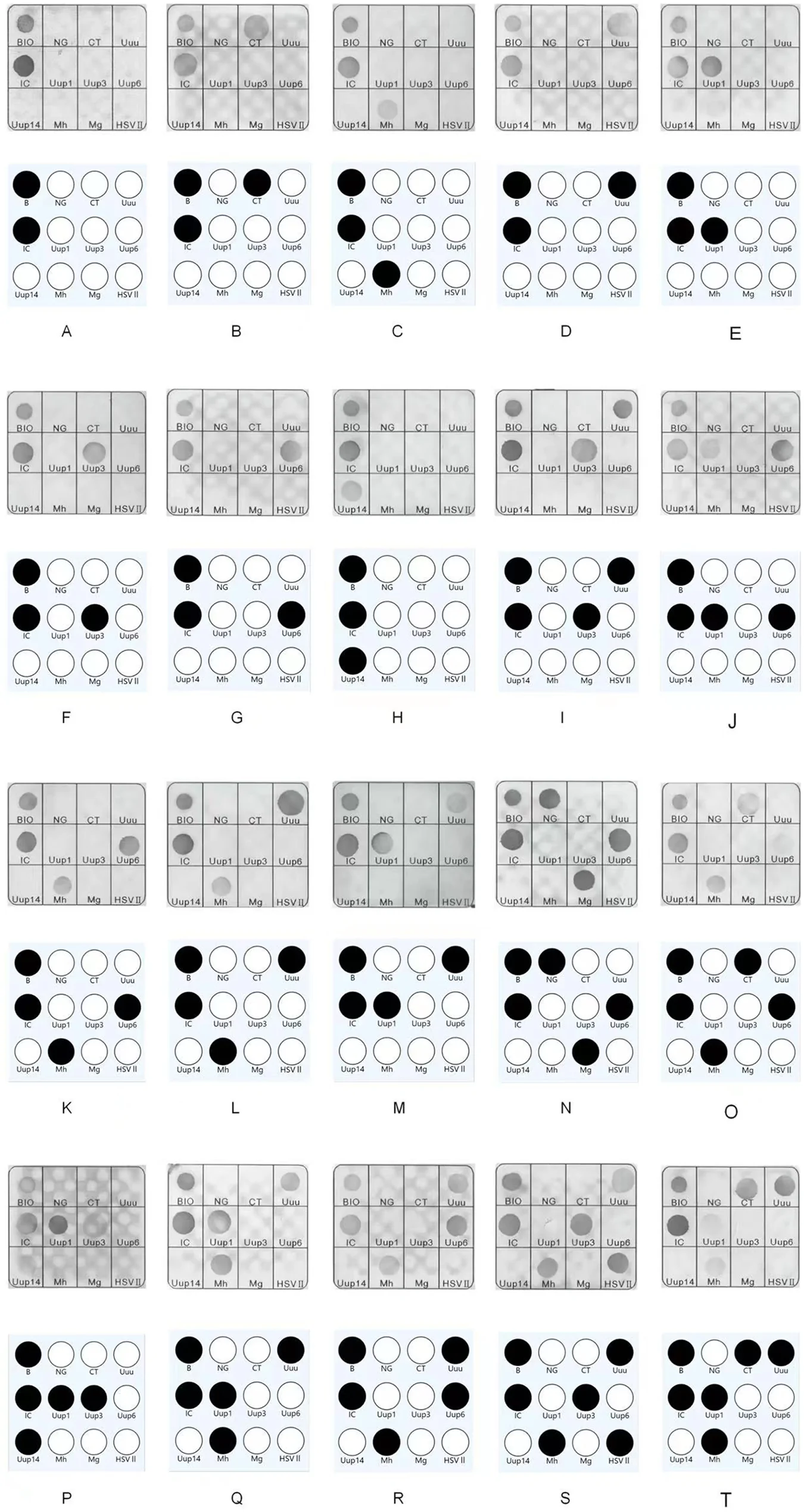
Representative detection patterns of genital pathogens **(A)** negative for reproductive tract pathogens; **(B)** positive for Chlamydia trachomatis (CT); **(C)** positive for Mycoplasma hominis (MH); **(D)** positive for Ureaplasma urealyticum (Uuu); **(E)** positive for Ureaplasma parvum serovar 1 (Uup1); **(F)** positive for Ureaplasma parvum serovar 3 (Uup3); **(G)** positive for Ureaplasma parvum serovar 6 (Uup6); **(H)** positive for Ureaplasma parvum serovar 14 (Uup14); **(I)** co-infection: Uuu and Uup3; **(J)** co-infection: Uup1 and Uup6; **(K)** co-infection: Uup6 and Mh; **(L)** co-infection: Uuu and Mh; **(M)** co-infection: Uup1 and Uuu; **(N)** co-infection: Uup6, Neisseria gonorrhoeae (NG), and Mycoplasma genitalium (MG); **(O)** co-infection: CT, Mh, and Uup6; **(P)** co-infection: Uup1, Uup3, and Uup14; **(Q)** co-infection: Uuu, Uup1, and Mh; **(R)** co-infection: Uuu, Uup6, and Mh; **(S)** co-infection: Uuu, Uup3, Mh, and HSV-2; **(T)** co-infection: Uuu, Uup1, Mh, and CT.

The results of non-HPV reproductive tract pathogen infections are determined by the flow-through hybridization spots on the biofilm. Figure 1 shows the interpretation of the results for the types of non-HPV reproductive tract pathogen infections.

In the HPV-positive group of 154 cases, high-risk and low-risk HPV types accounted for 84.4% (130/154) and 15.6% (24/154), respectively, with a genital tract infection pathogen detection rate of 62.3% (96/154). In contrast, the HPV-negative group of 193 cases exhibited a genital tract infection pathogen detection rate of 57.0% (110/193). The six most prevalent HPV subtypes were HPV16, 53, 52, 58, 51, and 33, with detection rates of 31.8%, 13.6%, 13.6%, 11.0%, 4.5%, and 1.9%, respectively. The genital tract infection pathogens, listed in descending order of detection rate, were Uup3, Uup6, Uuu, MH, Uup1, CT, Uup14, HSV-2, MG, and NG, with detection rates of 17.6%, 15.3%, 14.1%, 10.4%, 8.1%, 6.1%, 5.2%, 1.7%, 1.2%, and 0%, respectively. The detection rates for single, dual, and multiple pathogens in reproductive tract infections were 37.5% (130/347), 27.4% (95/347), and 14.7% (51/347), respectively, Table 1.

**Table 1:** Detection patterns and frequencies of genital pathogens in the study population (*n* = 347).

Pathogen	Single-pathogen detection (%)	Dual-pathogen detection (%)	Multiple-pathogen detection (%)	Total	Percentage (%)
Uuu	22	19	8	49	14.1
Uup1	15	8	5	28	8.1
Uup3	32	18	11	61	17.6
Uup6	29	15	9	53	15.3
Uup14	1	12	5	18	5.2
MH	7	21	8	36	10.4
MG	3	1	0	4	1.2
CT	18	0	3	21	6.1
HSV-2	3	1	2	6	1.7
HPV					
HR-HPV	52	15	11	78	50.6
LR-HPV	10	0	1	11	7.1

### 3.2 Associations between non-HPV genital pathogens and HPV positivity

**Figure 2:**
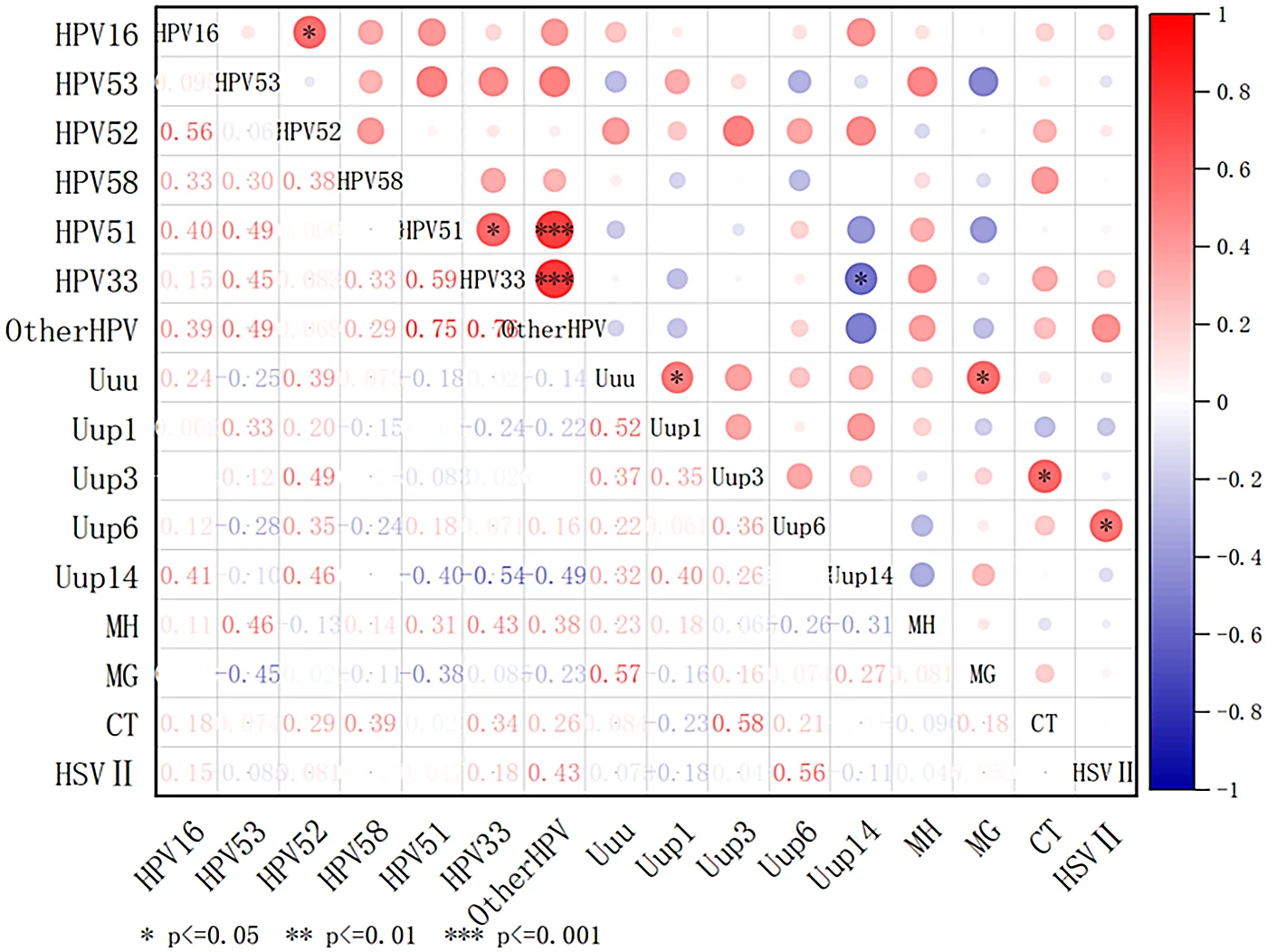
Spearman correlation analysis among reproductive tract pathogens. CT, chlamydia trachomatis; NG, Neisseria gonorrhoeae; UU, Ureaplasma urealyticum; MH, Mycoplasma hominis; Mg, Mycoplasma genitalium; HSV-2, Herpes simplex virus type II.

Since no NG-positive cases were detected, NG was excluded from the inferential analyses. HPV genotypes with small numbers of cases were combined into an “other HPV” group for further analysis. Spearman correlation analysis identified several statistically significant pairwise associations among HPV genotypes and genital pathogens. Specifically, HPV16 was positively correlated with HPV52 (*ρ* = 0.56, *P* < 0.05), and HPV33 was positively correlated with HPV51 (*ρ* = 0.59, *P* < 0.05). The “other HPV” group showed positive correlations with HPV33 and HPV51 (*ρ* = 0.75 and 0.76, respectively; *P* < 0.001). Among genital pathogens, Uuu was positively correlated with Uup1 (*ρ* = 0.52, *P* < 0.05) and MG (*ρ* = 0.57, *P* < 0.05); Uup3 was positively correlated with CT (*ρ* = 0.58, *P* < 0.05); and Uup6 was positively correlated with HSV-2 (*ρ* = 0.56, *P* < 0.05). An inverse correlation was observed between HPV33 and Uup14 (*ρ* = −0.54, *P* < 0.05). The results are shown in Figure 2.

No statistically significant differences in HPV positivity were observed between pathogen-negative and pathogen-positive participants for Uup3, Uup14, or HSV−2 (all *P* > 0.05). NG was not included in the inferential analysis because no NG-positive cases were detected. In contrast, statistically significant differences in HPV positivity were observed between pathogen-negative and pathogen-positive participants for Uuu, Uup1, Uup6, MH, and CT (*P* < 0.05), as shown in Table 2. Although MG showed a relatively large estimated OR (7.163), only four MG-positive cases were detected and the corresponding 95% CI was extremely wide and included 1.0 (95% CI: 0.734–69.898). Therefore, the MG estimate was considered imprecise and was not interpreted as reliable evidence of an association with HPV positivity. Overall, detection of Uuu, Uup1, Uup6, MH, and CT was statistically associated with HPV positivity, whereas Uup3, Uup14, and HSV-2 showed no statistically significant associations. The evidence for MG was inconclusive because of the very small number of positive cases.

**Table 2:** Differences in HPV positivity between pathogen-negative and pathogen-positive participants.

Pathogen	Negative or Positive (−/+)	Number of cases(%)	HPV-positive	HPV-negative	OR	95%CI	*P*-value
Uuu (*n* = 295)	−	246 (83.4)	63 (25.6)	183 (74.4)	2.179	1.156–4.107	0.015
	+	49 (16.6)	21 (42.9)	28 (57.1)			
Uup1 (*n* = 272)	−	244 (89.7)	69 (28.3)	175 (71.7)	2.926	1.324–6.469	0.006
	+	28 (10.3)	15 (53.6)	13 (46.4)			
Uup3 (*n* = 273)	−	212 (77.7)	68 (32.1)	144 (67.9)	0.753	0.397–1.427	0.383
	+	61 (22.3)	16 (26.2)	45 (73.8)			
Uup6 (*n* = 304)	−	251 (82.6)	60 (23.9)	191 (76.1)	2.634	1.426–4.867	0.002
	+	53 (17.4)	24 (45.3)	29 (54.7)			
Uup14 (*n* = 287)	−	269 (93.7)	79 (29.4)	190 (70.6)	0.925	0.319–2.681	0.886
	+	18 (6.3)	5 (27.8)	13 (72.2)			
MH (*n* = 283)	−	247 (87.3)	60 (24.3)	187 (75.7)	8.103	3.696–17.768	<0.05
	+	36 (12.7)	26 (72.2)	10 (27.8)			
MG (*n* = 275)	−	271 (98.5)	80 (29.5)	191 (70.5)	7.163	0.734–69.898	0.049
	+	4 (1.5)	3 (75.0)	1 (25.0)			
CT (*n* = 292)	−	271 (92.8)	80 (29.5)	191 (70.5)	47.75	6.301–361.852	<0.05
	+	21 (7.2)	20 (95.2)	1 (4.8)			
HSV-2 (*n* = 275)	−	269 (97.8)	81 (30.1)	188 (69.9)	1.160	0.208–6.463	0.865
	+	6(2.2)	2(33.3)	4(66.7)			

NG was excluded from inferential analysis because no NG-positive cases were detected. MG and HSV-2 were detected in only 4 and 6 participants, respectively; therefore, their OR estimates are based on sparse data and should be interpreted with caution. The wide 95% CIs indicate limited precision and statistical power. ORs with 95% CIs including 1.0 were not interpreted as statistically significant associations.

### 3.3 Detection of major genital pathogens across cervical cytological categories

**Figure 3:**
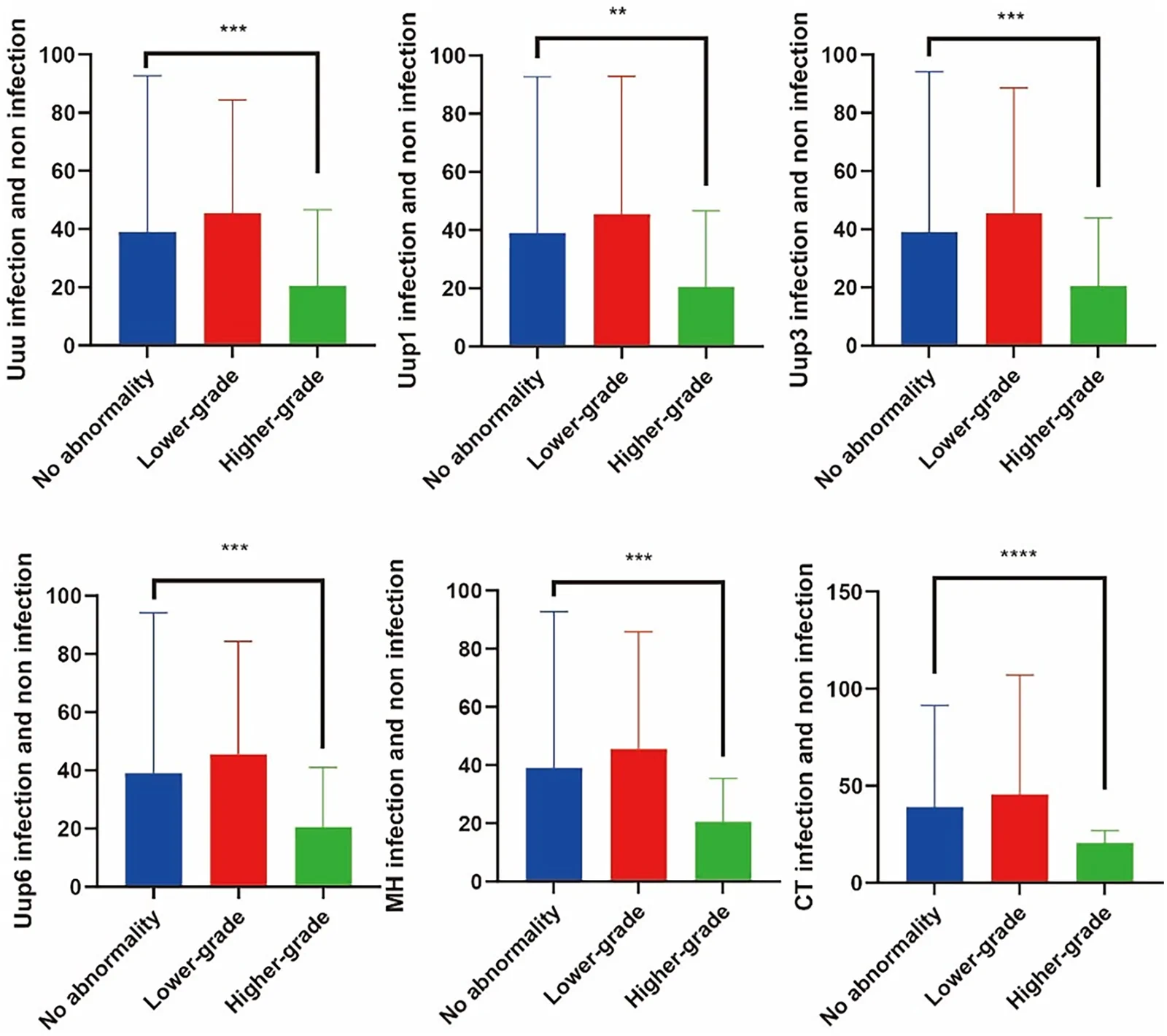
Detection of six genital pathogens across cervical cytological categories. CT, chlamydia trachomatis; UU, Ureaplasma urealyticum; MH, Mycoplasma hominis; LSIL, low-grade cervical cytological lesion; HSIL, high-grade cervical cytological lesion.

Among the 347 women with TCT results, 132 had cervical cytological abnormalities, including 91 (26.2%) in the lower-grade cervical cytological abnormality group and 41 (11.8%) in the higher-grade cervical cytological abnormality group. Because NG, MG, Uup14, and HSV-2 were detected in very few participants, the analyses across cervical cytological categories were restricted to the remaining six pathogens. The detection rates of Uuu, Uup1, Uup3, Uup6, MH, and CT differed significantly across the three cervical cytological categories: no epithelial cell abnormality, lower-grade cervical cytological abnormalities, and higher-grade cervical cytological abnormalities (*χ*^2^ = 17.454, 9.368, 13.902, 16.751, 16.393, and 51.462, respectively; all *P* < 0.05). Thus, detection of these six pathogens was statistically associated with cervical cytological abnormalities in this study population (Figure 3).

### 3.4 Association between genital pathogen detection and cervical histopathological findings

Among the 154 HPV-positive women, 124 underwent histopathological examination. The histopathological findings included 6 normal cases, 36 cases with inflammatory changes, and 82 cases with cervical SILs. Genital pathogens were detected in 83.3% (5/6) of the normal group, 58.3% (21/36) of the inflammatory group, and 54.9% (45/82) of the SIL group. Compared with the normal histopathology group, no statistically significant difference in genital pathogen detection was observed in either the inflammatory group (*P* = 0.243) or the SIL group (*P* = 0.174). Therefore, among HPV-positive women with available histopathological results, overall genital pathogen detection was not significantly associated with histopathological category. Refer to Table 3 for further details.

**Table 3:** Association between genital pathogen detection and cervical histopathological findings among HPV-positive women with available histopathology (*n* = 124).

Pathological results	Pathogens of genital tract infection		OR	95%CI	*P*-value
	Negative (%)	Positive (%)			
Normal group (*n* = 6)	1 (16.7)	5 (83.3)			
Inflammation group (*n* = 36)	15 (41.7)	21 (58.3)	3.571	0.378–33.782	0.243
SIL group (*n* = 82)	37 (45.1)	45 (54.9)	4.111	0.460–36.757	0.174
Total number (*n* = 124)	53 (42.7)	71 (57.3)			

## 4 Discussion

The present retrospective cross-sectional study examined the associations between non-HPV genital pathogen detection, HPV positivity, cervical cytological abnormalities, and histopathological findings in women undergoing cervical cancer screening in Xinjiang. Several observations were documented. First, Uup3, Uup6, and Uuu were among the most frequently detected non-HPV genital pathogens in this study population. Second, detection of Uuu, Uup1, Uup6, MH, and CT was associated with HPV positivity, whereas Uup3, Uup14, and HSV-2 showed no statistically significant association with HPV positivity. The estimate for MG was considered inconclusive because of the very small number of positive cases and the wide 95% confidence interval. Third, detection of Uuu, Uup1, Uup3, Uup6, MH, and CT differed across cervical cytological categories. Importantly, however, among HPV-positive women with available histopathological findings, overall genital pathogen detection was not significantly associated with histopathological category. These findings suggest associations between selected genital pathogens, HPV positivity, and concurrent abnormal cervical cytology, but they do not establish that these pathogens cause HPV infection or promote histopathological progression (10).

Although HPV and non-HPV genital pathogens represent distinct microbial entities, both may share sexual transmission routes (11). Co-detection of HPV and other genital pathogens has been reported in women with HPV-related cervical lesions, and observational studies have suggested associations between such co-detection and cervical disease (12). Previous studies have also reported associations between Uup6, MH, MG, and CT detection and HPV positivity (13). Previous studies have reported an association between HPV and *Chlamydia trachomatis* co-detection/co-infection in relation to cervical neoplasia, although the temporal and causal relationships remain uncertain (14). Associations between selected genital pathogens and high-grade cervical abnormalities have also been reported in previous studies (15). HSV-2 positivity has similarly been investigated in relation to HPV persistence and precancerous cervical abnormalities (15). Associations of CT and BV with HPV-related cervical lesions have been investigated for many years (16–18). In contrast, the potential role of *Ureaplasma* spp. as a cofactor in HPV positivity and cervical abnormalities has received increasing attention more recently. In a multicenter molecular epidemiological study, Valasoulis et al. reported that bacterial sexually transmitted pathogen positivity was associated with HPV positivity and abnormal cervical cytology, with *Ureaplasma* spp. among the most frequently detected organisms (19). These findings add to the emerging evidence that Ureaplasma spp. detection may be associated with HPV positivity and abnormal cervical cytology; however, its causal role in HPV persistence or cervical lesion progression remains uncertain. At the molecular level, HPV-associated cervical carcinogenesis involves alterations in host cellular pathways and cancer-related molecular processes (20, 21).

It should also be noted that molecular detection of genital pathogens does not necessarily indicate clinically active infection. Particularly for organisms that may be detected in asymptomatic women, such as Ureaplasma spp. and M. hominis, molecular positivity may in some cases represent colonization or asymptomatic carriage rather than active infection. Accordingly, the associations observed in the present study are interpreted in terms of pathogen detection or positivity rather than clinically relevant active infection.

In the present study population from Xinjiang, Uup3 and Uup6 were the most frequently detected non-HPV genital pathogens, with detection rates of 17.6% and 15.3%, respectively, followed by Uuu (14.1%), MH (10.4%), Uup1 (8.1%), and CT (6.1%). These findings were partly comparable with those reported in Shanghai. Xie et al. examined 668 women using a similar ten-pathogen panel and flow-through hybridization method and reported detection rates of 19.16% for Uup3, 15.72% for Uup6, 11.83% for Uuu, 17.07% for MH, 9.88% for Uup1, and 9.43% for CT (13). Thus, Uup3 and Uup6 showed similar detection frequencies in the two study populations, whereas MH and CT were less frequently detected in our Xinjiang cohort. Regarding HPV genotype distribution, HPV16 was the most frequently detected genotype among HPV-positive participants in our study, followed by HPV53, HPV52, and HPV58. In contrast, a large cervical screening study involving 198,111 women in Guangzhou reported HPV52 as the most prevalent genotype, followed by HPV16 and HPV58 (22). Therefore, HPV16, HPV52, and HPV58 were common genotypes in both populations, although their relative rankings differed. Internationally, a multicenter study of 2,256 women in Greece reported that bacterial genital pathogen positivity was associated with HPV positivity and that bacterial pathogens were more frequently detected among women with LSIL than among those with normal cervical cytology (19). These observations are broadly consistent with the associations between genital pathogen detection, HPV positivity, and cervical cytological abnormalities observed in our study. Nevertheless, comparisons across studies should be interpreted cautiously because of differences in participant characteristics, recruitment settings, pathogen panels, laboratory methods, and other population characteristics. Therefore, our findings provide regional clinical data from Xinjiang but should not be interpreted as defining a distinct epidemiological profile for the entire Xinjiang population.

The temporal relationship between cervical epithelial abnormalities, HPV positivity, and detection of non-HPV genital pathogens remains uncertain. Changes in the vaginal and cervical microbial environment have been proposed to influence local inflammatory and immune responses, which may be associated with HPV persistence and cervical epithelial abnormalities (23). Conversely, cervical epithelial changes, local inflammation, and impairment of epithelial barrier function may also alter the cervical microenvironment and facilitate the persistence or detection of other genital microorganisms. Therefore, the relationship may be bidirectional. Because HPV status, genital pathogen detection, and cervical abnormalities were evaluated cross-sectionally in the present study, the temporal sequence of these events cannot be established.

From a clinical perspective, established approaches to cervical cancer prevention and management are based on cervical screening and evidence-based management of abnormal cervical findings, with treatment strategies guided by disease stage and clinical context (24–26). Regional HPV epidemiology also remains relevant when interpreting HPV genotype distributions and screening populations (22). In parallel, increasing attention has been directed toward the interactions among HPV, other sexually transmitted pathogens, and the genital microbiome, as well as their potential effects on the cervical microenvironment (27, 28). However, the present cross-sectional findings do not demonstrate that broad genital pathogen screening improves cervical cancer outcomes or should modify established cervical screening or management algorithms. Rather, the observed associations should be regarded as hypothesis-generating and provide a basis for prospective studies evaluating whether pathogen-specific assessment offers clinically meaningful information beyond established HPV-based approaches.

Importantly, the cytological and histopathological findings in the present study should be interpreted separately. The detection rates of Uuu, Uup1, Uup3, Uup6, MH, and CT differed significantly across the cervical cytological categories, indicating associations between these pathogens and concurrent cytological abnormalities. In contrast, among HPV-positive women with available histopathological results, overall genital pathogen detection did not differ significantly between the normal histopathology group and either the inflammatory group (*P* = 0.243) or the SIL group (*P* = 0.174). Therefore, our data do not support the conclusion that non-HPV genital pathogen detection is associated with increasing histopathological severity. Several factors may contribute to this negative finding. The histopathologically normal group included only six women, resulting in limited statistical power and imprecise effect estimates. Therefore, the absence of statistical significance should not be interpreted as definitive evidence of the absence of an association. In addition, the broad grouping of heterogeneous histopathological abnormalities may have obscured associations with specific histopathological categories. It is also possible that some genital pathogens are more closely associated with HPV positivity or concurrent cytological abnormalities than with histopathological progression. Prospective studies with larger biopsy cohorts, longitudinal HPV and pathogen testing, and more detailed histopathological stratification are needed to examine these possibilities.

From a therapeutic perspective, the associations observed in this study should be interpreted cautiously. Because no therapeutic intervention or longitudinal follow-up was performed, the present findings do not demonstrate that treatment of non-HPV genital pathogens promotes HPV clearance or prevents cervical lesion progression. Nevertheless, detection of clinically relevant genital pathogens during cervical screening may prompt further clinical evaluation, and confirmed active infections should be managed according to established clinical indications. Molecular positivity alone should not be considered an indication for empirical antimicrobial treatment, particularly for organisms for which colonization or asymptomatic carriage may occur, especially in the absence of symptoms or abnormal cytology. Future prospective interventional studies are needed to determine whether pathogen-specific treatment influences HPV persistence, viral clearance, cervical cytological abnormalities, or histopathological outcomes.

This study has several limitations. First, it was a single-center retrospective cross-sectional study with a relatively limited sample size, which may restrict the generalizability of the findings. Because all participants were recruited from a single clinical center in Urumqi, the observed genital pathogen and HPV genotype distributions should not be considered representative of the entire Xinjiang population. Second, the cross-sectional design does not establish the temporal sequence between genital pathogen detection, HPV positivity, and cervical abnormalities. Therefore, the observed associations cannot be interpreted as evidence that non-HPV genital pathogens cause HPV infection or promote cervical lesion progression (29). Third, the histopathological analysis included only 124 HPV-positive women, and the normal histopathology group contained only six participants, resulting in limited statistical power. Fourth, several pathogens were detected in relatively small numbers, particularly MG and HSV-2, while NG was not detected, limiting the precision of pathogen-specific estimates. In addition, the molecular assay provided qualitative pathogen detection but did not assess pathogen load, clinical symptoms, culture results, or longitudinal persistence. Therefore, pathogen positivity could not be equated with clinically active infection, and colonization or asymptomatic carriage cannot be excluded for organisms for which such states are biologically plausible. Detailed information on several potential confounding factors was not available, including sexual behavior beyond a history of sexual intercourse, parity, contraceptive use, smoking status, HPV vaccination status, and other host-related or immune-related characteristics. Consequently, these variables could not be included in multivariable adjustment, and residual confounding may have influenced the observed associations.

In conclusion, this retrospective cross-sectional study showed that detection of Uuu, Uup1, Uup6, MH, and CT was associated with HPV positivity in women undergoing cervical cancer screening in Xinjiang. Detection of selected genital pathogens also differed across cervical cytological categories, whereas no statistically significant association was identified between overall genital pathogen detection and histopathological category among HPV-positive women. These findings indicate associations between genital pathogen detection, HPV positivity, and concurrent cervical cytological abnormalities, but do not establish causal relationships or demonstrate that these pathogens promote cervical lesion progression. Larger multicenter prospective studies with longitudinal follow-up are needed to validate these findings and clarify their temporal and clinical significance. Future studies should incorporate quantitative pathogen measurements, repeated HPV and pathogen testing, detailed histopathological grading, distinction between active infection and colonization where appropriate, and comprehensive assessment of potential confounding factors to determine whether these associations have implications for cervical screening and pathogen-specific clinical management.

## Data Availability

The original contributions presented in the study are included in the article/supplementary material, further inquiries can be directed to the corresponding author/s.
